# The epidemiology and outcomes of adults with acute hypoxaemic respiratory failure in a low-income country in the context of the COVID-19 pandemic: a prospective, observational, multicentre cohort study

**DOI:** 10.1136/bmjgh-2024-017949

**Published:** 2025-08-17

**Authors:** Arthur Kwizera, Daphne Kabatoro, Cornelius Sendagire, Jane Nakibuuka, Darius Owachi, Christopher Nsereko, John Paul Ochieng, Maria Goretti Nampiina, Mary Jane Nampaawu, Dennis Kakaire, Morris Baluku, Eric Odwar, George Kateregga, Martin Duenser, Charles Olaro, Henry Kyobe-Bosa, Bruce J Kirenga, Lydia Nakiyingi, Pauline Byakika-Kibwika, Noah Kiwanuka, David Patrick Kateete, Moses Joloba, Charlotte Summers, for the ARISE-Uganda Investigators

**Affiliations:** 1 Anaesthesia and Critical Care, Makerere University, Kampala, Uganda; 2 Makerere University, Kampala, Uganda; 3 Ministry of Health, Brasilia, Brazil; 4 Makerere University College of Health Sciences, Kampala, Uganda; 5 Entebbe General Hospital, Entebbe, Uganda; 6 Mbale Regional Referral Hospital, Mbale, Uganda; 7 Mengo Hospital, Kampala, Uganda; 8 Rubaga Hospital, Kampala, Uganda; 9 Masaka Regional Referral Hospital, Masaka, Uganda; 10 Kabale Regional Referral Hospital, Kabale, Uganda; 11 Lacor Hospital, Gulu, Uganda; 12 Mbarara National Referral Hospital, Mbarara, Uganda; 13 Department of Anaesthesiology and Intensive Care Medicine, Kepler University Hospital, Linz, Austria; 14 Director General Health Services, Republic of Uganda Ministry of Health, Kampala, Uganda; 15 Republic of Uganda Ministry of Health, Kampala, Uganda; 16 University of Oxford Kellogg College, Oxford, UK; 17 Internal Medicine, Makerere University Faculty of Medicine, Kampala, Uganda; 18 School of Public Heatlh, Makerere University College of Health Sciences, Kampala, Uganda; 19 Makerere University School of Health Sciences, Kampala, Uganda; 20 Medical Microbiology, Makerere University College of Health Sciences, Kampala, Uganda; 21 Victor Phillip Dahdaleh Heart and Lung Research Institute, University of Cambridge, Cambridge, UK

**Keywords:** Epidemiology, Cohort study, Health systems

## Abstract

**Background:**

Few data regarding the incidence and outcomes of acute hypoxaemic respiratory failure (AHRF) in low- and middle-income countries exist.

**Methods:**

We undertook a prospective, observational multicentre study at 11 Ugandan hospitals (July 2020–April 2021) to determine the prevalence, aetiology and 28-day all-cause mortality of AHRF (acute shortness of breath plus peripheral oxygen saturation <91% while breathing ambient air) in adults (≥18 years) who required unplanned hospitalisation.

**Findings:**

16 747 adults required unplanned hospitalisation during the study period. The median age of study participants was 50 years, and 65.1% were male. The prevalence of AHRF was 4.1%. The predominant causes were pulmonary (46.8%) and extrapulmonary infection (18.3%). Only 38 patients (5.6%) received invasive mechanical ventilation. All-cause mortality 28 days after hospitalisation was 37.9% and associated with the severity of hypoxaemia at presentation (p<0.001). Risk factors for death included oxygen saturation (adjusted relative risk (aRR) 0.96 (95% CI 0.93 to 0.98); p=0.001), the lung injury prediction score (aRR 1.83 (95% CI 1.43 to 2.36); p<0.001), respiratory rate>30 breaths per minute (aRR 2.39 (95% CI 1.34 to 4.26); p=0.003) and age >65 years (aRR 2.09 (95% CI 1.13 to 2.86); p=0.02).

**Interpretation:**

In the context of the COVID-19 pandemic, the prevalence of AHRF among adults requiring unplanned hospitalisation in Uganda was comparable with that reported by previous single-centre studies. Pulmonary infection was the most common cause of AHRF. The high 28-day mortality may be explained by the severity of the disease at presentation and the limited access to advanced organ support, including invasive mechanical ventilation.

**WHAT IS ALREADY KNOWN ON THIS TOPIC**

- Acute hypoxaemic respiratory failure (AHRF) is a common cause of hospital admission, critical illness and death around the globe.
- The majority of the published literature on the prevalence, management and clinical outcomes of AHRF originates from high-income countries.
- Few data from low- and middle-income countries (LMICs) are available.
- Our previous single-centre study conducted in Uganda reported that the incidence of AHRF was low (4.5%) in patients presenting to hospital, but AHRF was associated with a 90-day mortality of 85%.

**WHAT THIS STUDY ADDS**

- We undertook a prospective, observational multicentre study at 11 hospitals across Uganda and observed a 4.1% incidence of AHRF among adults requiring unplanned hospitalisation.
- Pulmonary (COVID-19, non-COVID lower respiratory tract infections and tuberculosis) and extrapulmonary infections were the most common causes of AHRF.
- Non-communicable disease-associated AHRF constituted most of the remaining cases.
- The 28-day all-cause mortality associated with AHRF was 37.9% and may be explained by both the high-disease severity at presentation as well as limited access to advanced organ support such as invasive mechanical ventilation.

**HOW THIS STUDY MIGHT AFFECT RESEARCH, PRACTICE OR POLICY**

- Our data illustrate the burden (prevalence, mortality and factors associated with increased mortality) and causes of AHRF in a sub-Saharan setting.
- These data, including the high 28-day mortality, may assist healthcare stakeholders and policymakers when planning for the future development of hospital and critical care services/infrastructure in LMICs.
- They may also help to inform guidelines to improve the management of AHRF and identification of those with the highest risk of death.

## Introduction

 Acute hypoxaemic respiratory failure (AHRF) is a common cause of hospital admission, critical illness and death across the globe.^1^,^3^ The majority of the published literature regarding the prevalence, management and clinical outcomes of AHRF originates from high-income countries,^2 3^ with little published data from low- and middle-income countries (LMICs). The COVID-19 pandemic has highlighted the wide gap between the burden of AHRF and the capacity of healthcare systems to manage this condition in many areas of the world, particularly LMIC settings. A recent African multicentre, prospective, observational cohort study revealed that the mortality of critically ill patients with COVID-19 was higher in African countries than in Asia, Europe,^4^ North America and South America. This observation was attributed to insufficient critical care resource, as well as the burden of comorbidities such as HIV/AIDS and chronic non-communicable diseases.^5^

Previously, (November 2015–July 2017), our group (ARISE-Africa) conducted a prospective, single-centre observational study at the Mulago National Referral Hospital in Kampala, Uganda to define the incidence and outcome of patients with AHRF. We observed that the prevalence of AHRF was modest (4.5%), but AHRF was associated with an excessively high 90-day mortality of 85%, in a population where the median age was 38 years.^1^ Here, we present a prospective, observational, multicentre cohort study from 11 referral hospitals across Uganda to determine the epidemiology and outcome of AHRF among adults requiring unplanned hospital admission.

## Methods

### Study design

**Figure 1:**
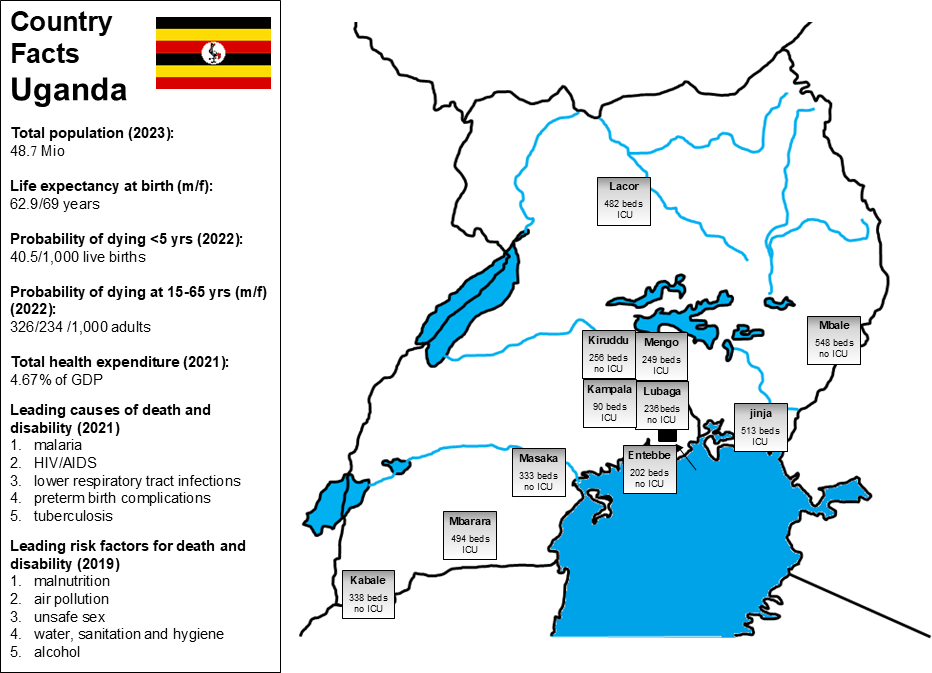
Country information for Uganda, overview of study sites. GDP, gross domestic product; ICU, intensive care unit; Mio, million; pop., population; yrs, years.

This prospective, observational, multicentre national cohort study was conducted at 11 public hospitals in Uganda (figure 1) between 1 July 2020 and 30 April 2021. Hospital sites were recruited through the Acute Respiratory Interventions (ARISE-Africa) group. The article was prepared in accordance with the updated Strengthening the Reporting of Observational Studies in Epidemiology checklist for reporting cohort studies.^6^

### Patient selection

All adult (≥18 years) patients who required unplanned hospitalisation in one of the study hospitals were screened for study eligibility. Patients who presented with AHRF or who developed AHRF during the first 24 hours following hospital admission were included in the study. Patients admitted in a moribund state (as determined by the attending physician), and subjects, in whom written informed consent could not be obtained, were excluded. In line with the definition applied in a previous observational study conducted in Uganda, AHRF was defined as acute (<7 days) shortness of breath combined with a peripheral pulse oximetry reading <91% while breathing ambient air (or a plethysmographic oxygen saturation to the fraction of inspired oxygen ratio of <316 in patients who had already received oxygen).

### Study setting and management of AHRF

The study was conducted during Uganda’s first COVID-19 wave. In response to the COVID-19 pandemic, the Ugandan government supplied all hospitals, including the 11 study sites, with staff training, oxygen and oxygen administration consumables to increase the capacity to manage the additional healthcare burden imposed by the COVID-19 pandemic. All patients with fever and/or acute respiratory symptoms were systematically screened for the presence of SARS-CoV-2 using nasopharyngeal swabs.

Specimens were sent to regional laboratories for PCR testing, and results were usually reported within 24–48 hours of collection. In case of hypoxaemia, oxygen was, wherever available, applied through nasal prongs or a face mask with a reservoir bag and titrated to reach a peripheral plethysmographic oxygen saturation ≥91%. There was limited access to non-invasive ventilation and high-flow oxygen devices. 4 of the 11 study hospitals ran a dedicated intensive care service and were able to provide mechanical ventilation. No invasive respiratory organ support other than mechanical ventilation was offered. Laboratory and radiological (including chest radiographs and bedside sonography) capacities were limited at all study sites. Patients, who presented to a study hospital without an intensive care unit but who required mechanical ventilation, were referred to a healthcare facility with an intensive care unit whenever possible, and when bed availability allowed. According to the National COVID-19 treatment guidelines, patients with COVID-19 requiring advanced respiratory support were directly referred from health centres or primary care hospitals to the national severe COVID-19 centre at the Mulago Hospital in Kampala. Thus, patients with severe AHRF due to COVID-19 were not referred to any of the 11 study sites if they had first presented to another healthcare facility.

The treatment of patients with lower respiratory tract infections (LRTIs) and COVID-19 was based on national treatment guidelines. This, for example, included monitoring of the plethysmographic oxygen saturation with a pulse oximeter, oxygen administration when necessary and empirical use of intravenous antibiotics in adults who require hospitalisation because of suspected community-acquired pneumonia from bacteria.^7^ In adults with moderate or severe pneumonia due to infection with the SARS-CoV-2 virus, the contemporary National Guidelines for the Management of COVID-19 (version April 2020) recommended the use of oral azithromycin (at daily doses of 500 mg for 5 days) or amoxicillin (at daily doses of 500 mg 8 per hour for 1 week), oral hydroxychloroquine (at a dose of 400 mg 12 per hour on day 1, followed by 200 mg 12 per hour on days 2–5), oral vitamin C (at daily doses of 500 mg 12 per hour for 14 days) and oral zinc (at daily doses of 20 mg per day for 14 days).^7 8^

### Data collection

At each site, all study-related data were collected by dedicated research staff, who screened patients in the emergency department of each study site as well as within 24 hours after hospital admission and followed study subjects throughout their hospital stay. None of the research personnel was involved in the clinical care of any study patient. A standardised case report form was used for data documentation (Electronic online supplemental file 1). Data collection occurred at the following time points (and hospital sites): study inclusion (emergency department or patient receiving areas as well as follow-up visit within 24 hours on the hospital ward), hospital discharge (follow-up visits on the ward) and day 28 after study inclusion (follow-up visits on the ward or by telephone contact).

The following data were collected at study enrolment: age, sex, comorbid conditions, history of smoking, distance from home to the hospital of admission, source of admission (community vs referral from another hospital), cause of AHRF, vital parameters (heart rate, respiratory rate, mean blood pressure and plethysmographic oxygen saturation), type of oxygen administration device used and the fraction of inspired oxygen (estimated from oxygen flow rates and oxygen application devices as suggested by the conversion tables from the Extended Prevalence of Infection in Intensive Care II study).^9^ The ratio between the plethysmographic oxygen saturation and the fraction of inspired oxygen was calculated. The plethysmographic oxygen saturation was measured with the use of commercially available pulse oximeters at each study site. No further calibrations of these devices were performed for the reason of this study. In addition, the following scores were calculated using data collected at study enrolment: the Universal Vitals Assessments Score,^10^ the CURB65 Score^11^ and the Lung Injury Prediction Score.^12^ In view of the inconsistent access to radiography and sonography machines, we could not determine the prevalence of acute respiratory distress syndrome (ARDS) in this study cohort.^13^

Throughout the hospital stay and at hospital discharge, the need for ICU admission and use of invasive mechanical ventilation, as well as the length of hospital stay, were documented. Survival status was determined at day 28 after study inclusion. If patients were discharged from the hospital before day 28, research staff contacted the study patient or the next of kin using a telephone number documented while collecting informed consent.

### Definitions

Infectious causes of AHRF were categorised as COVID-19, LRTIs other than COVID-19 or extrapulmonary infections. COVID-19 was defined as an infection caused by the SARS-CoV-2 virus detected by either an antigen or PCR test. Extrapulmonary infections were defined as infections originating in organs other than the lungs. These included, for example, malaria, bloodstream infections, skin or abdominal infections. Based on the ratio between the plethysmographic oxygen saturation and the fraction of inspired oxygen, the severity of hypoxaemia was categorised into four grades: ≥316 (no hypoxaemia), 236–315 (mild hypoxaemia), 149–235 (moderate hypoxaemia) and ≤148 (severe hypoxaemia).^14^

### Outcomes

The primary outcomes were the prevalence, aetiology and 28-day all-cause mortality associated with AHRF. The secondary study outcome was to define the risk factors for death from AHRF.

### Sample size estimation

We did not perform a formal power calculation prior to the onset of this observational cohort study. However, based on the results of a previous prospective observational study, which was conducted at one Ugandan tertiary referral hospital over 21 months that reported a prevalence of AHRF among critically ill adults of 4.5%,^1^ we assumed that the data collection over a period of 10 months at 11 hospitals in Uganda would allow the inclusion of an adequate number of patients (at least 500) to reliably estimate the prevalence, aetiology and 28-day mortality rate associated with AHRF in Uganda.

### Data anonymisation and processing

Data were collected on paper case report forms. Before the entry of study variables into a dedicated electronic database, a numeric code was used to pseudoanonymise all data. During the study, all paper case report forms containing identifiable patient data were kept in a locked room or cupboard at each study site. After completion of the study, all case report forms were transferred to the main study site, where they were stored in an archive according to the national recommendations. All data were double entered into the electronic database. After completing data entry into the electronic database, the database was locked and quality control checks were performed to identify entry or documentation errors. Wherever possible, these entry or documentation errors were rectified, and missing data were retrieved from patient charts.

The data analysis used the Stata/IC 14.2 (StataCorp; College Station, Texas, USA). Descriptive statistical methods were used to report demographic, clinical, risk score and outcome data. Hospital survivors and non-survivors were compared using the Mann–Whitney U rank sum, χ² or Fisher’s exact test, as appropriate. Kaplan–Meier plots were drawn for the entire study population and for study patients categorised by the ratio between the plethysmographic oxygen saturation and the fraction of inspired oxygen. The log-rank test was used to compare mortality between patients within the four categories defined above. A modified *Poisson* regression model was fitted using a log link to identify independent risk factors for 28-day mortality. All variables that significantly differ between survivors and non-survivors at day 28 were introduced into the multivariate model, provided that there was no evidence of collinearity (assessed by variance inflation factor). We did not include therapeutic interventions such as mode of oxygen administration or intensive care admission, as these could only be applied in case of availability or capacity (eg, intensive care unit bed availability). A multiple imputation with chained equations method was used to impute missing values for variables, and ten imputed datasets were constructed. Finally, the model with the highest F-statistic was determined.

A *p*-value <0.05 was considered to indicate statistical significance. Data are presented as median values with IQRs or for categorical data absolute numbers with percentages, unless, otherwise indicated.

### Patient and public involvement

No patients or members of the public were involved in the design or delivery of this study.

### Role of the funding sources

The sponsors had no role in the study design, data collection, analysis, interpretation, manuscript drafting or decision to submit the manuscript for publication. The authors confirm that they have had full access to all the study data and take final responsibility for the decision to submit for publication.

## Results

**Figure 2:**
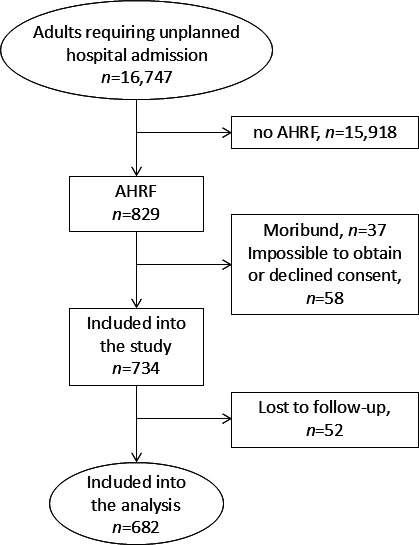
Study flow diagram. AHRF, acute hypoxaemic respiratory failure.

During the study period, 16 747 adults required unplanned hospitalisation at the 11 study sites. 682 subjects fulfilled all inclusion criteria and were enrolled into the study (figure 2). This corresponded to an AHRF prevalence of 4.1% (95% CI 3.8% to 4.4%) in study participants who required unplanned hospitalisation. AHRF was present in 466/682 (68.3%) participants at admission to hospital and developed during the first 24 hours of hospital admission in further 216 subjects (31.7%). 38 study participants (5.6%) were admitted to intensive care and received invasive mechanical ventilation during the course of their hospitalisation. Five patients (0.7%) were referred to another hospital to facilitate the increased levels of organ support (ie, invasive mechanical ventilation).

Table 1 summarises the demographic, epidemiological and outcome data from all study participants. At study entry, patients with AHRF who died within 28 days of hospital admission were older and presented with a higher respiratory rate, a lower mean arterial blood pressure and a lower plethysmographic oxygen saturation at study entry than those who survived. The degree of hypoxaemia was more pronounced, and both the lung injury prediction score and the universal vital assessment score were higher in those who did not survive 28 days. While survivors more often received oxygen via nasal prongs, in non- survivors, oxygen was more frequently administered using a face mask with a reservoir bag and non-invasive ventilation (table 2).

**Table 1:** Demographic and epidemiological characteristics of all study patients, survivors and non-survivors at day 28

		Total	Survivors	Non-survivors	P value
n		682	424	258	
Age	years	50 (40–63)	48 (38–60)	55 (41–67)	<0.001*
Age>65 years	n (%)	130 (19.1)	66 (15.6)	64 (24.8)	0.003*
Male sex	n (%)	444 (65.1)	275 (64.9)	169 (65.5)	0.86
Any comorbid condition	n (%)	425 (62.3)	262 (61.8)	163 (63.2)	0.75
Arterial hypertension	n (%)	153 (22.4)	94 (22.2)	59 (22.8)	0.85
HIV	n (%)	153 (22.4)	100 (23.6)	53 (20.6)	0.39
Diabetes mellitus	n (%)	59 (8.7)	42 (9.9)	17 (6.6)	0.16
Congestive heart failure	n (%)	20 (2.9)	14 (3.3)	6 (2.4)	0.64
Others	n (%)	40 (5.9)	12 (4.6)	28 (10.9)	<0.001*
History of smoking	n (%)	34 (5)	16 (3.8)	18 (7)	0.08
Distance from home to hospital of admission	km	10 (4–27)	10 (4–22)	10 (4–36)	0.26
Source of admission	n (%)				<0.001*
Community		376 (55.1)	275 (64.9)	101 (39.1)	
Referred from other hospital		306 (44.9)	149 (35.1)	157 (60.9)	
Cause of AHRF					
COVID-19	n (%)	199 (29.2)	149 (35.1)	50 (19.4)	<0.001*
Extrapulmonary infection	n (%)	125 (18.3)	68 (16)	57 (22.1)	0.05
LRTI (not COVID-19)	n (%)	103 (15.1)	60 (14.2)	43 (16.7)	0.38
Hypertensive emergency	n (%)	53 (7.8)	38 (9)	15 (5.8)	0.14
Diabetic emergency	n (%)	27 (4)	18 (4.2)	9 (3.5)	0.69
Acute/chronic renal failure	n (%)	24 (3.5)	12 (2.8)	12 (4.7)	0.28
Stroke	n (%)	21 (3.1)	10 (2.4)	11 (4.3)	0.18
Congestive heart failure	n (%)	19 (2.8)	12 (2.8)	7 (2.7)	>0.999
Tuberculosis	n (%)	17 (2.5)	8 (1.9)	9 (3.5)	0.21
Others	n (%)	94 (13.8)	49 (11.6)	45 (17.4)	0.04*

Continuous variables are presented as median values with IQRs, and categorical variables are numbers with percentages.

* Significant difference between survivors and non-survivors.

AHRF, acute hypoxaemic respiratory failure; LRTI, lower respiratory tract infection.

**Table 2:** Vital parameters, oxygen administration and risk score count at study entry of all study patients, survivors and non-survivors at day 28

		Total	Survivors	Non-survivors	P value
n		682	424	258	
Vital parameters					
Heart rate	breaths per minute	99 (87–112)	99 (87–112)	100 (87–117)	0.24
Respiratory rate	breaths per minute	25 (23–30)	25 (22–28)	27 (24–35)	<0.001*
Respiratory rate >30 breaths per minute	n (%)	145 (21.3)	68 (16)	77 (29.8)	<0.001*
Mean blood pressure	mm Hg	90 (73–103)	90 (80–103)	83 (65–102)	<0.001*
SpO_2_	%	88 (81–91)	88 (84–91)	86 (79–91)	0.002*
SpO_2_ ranges					
>90%	n (%)	188 (27.6)	119 (28.1)	69 (26.7)	0.72
90%–85%	n (%)	240 (35.2)	167 (39.4)	73 (28.3)	0.004*
<85%	n (%)	254 (37.2)	138 (32.5)	116 (45)	0.001*
Oxygen administration					
Nasal prongs	n (%)	250 (36.7)	216 (50.9)	34 (13.2)	<0.001*
Non-rebreather mask	n (%)	213 (31.2)	102 (24.1)	111 (43)	<0.001*
High-flow oxygen device	n (%)	10 (1.5)	6 (1.4)	4 (1.6)	>0.999
NIV	n (%)	71 (10.4)	16 (3.8)	55 (21.3)	<0.001*
No oxygen/missing	n (%)	138 (20.2)	84 (19.8)	54 (20.9)	0.77
SpO_2_/FiO_2_ ratio		171 (108–242)	215 (131–256)	113 (96–164)	0.002*
≥316	n (%)	12 (1.8)	10 (2.4)	2 (0.8)	0.15
200–315	n (%)	241 (35.3)	204 (48.1)	37 (14.3)	<0.001*
100–199	n (%)	258 (37.8)	140 (33)	118 (45.7)	0.001*
<100	n (%)	101 (14.8)	29 (6.8)	72 (27.9)	<0.001*
No oxygen/FiO_2_ missing	n (%)	70 (10.3)	41 (9.7)	29 (11.2)	0.52
Risk score counts					
CURB65	points	2 (1–3)	2 (1–3)	2 (1–3)	0.93
CURB65≥3 points	n (%)	131 (19.2)	89 (21)	42 (16.3)	>0.999
LIP score	points	3.5 (3–4.5)	3 (3–4)	4.5 (3.5–6)	0.001*
LIP score>4.5 points	n (%)	90 (13.2)	31 (7.3)	59 (22.9)	<0.001*
UVA score	points	3 (2–4)	2 (2–4)	3 (2–5)	<0.001*
UVA score≥5 points	n (%)	129 (18.9)	53 (12.5)	76 (29.5)	<0.001*

Continuous variables are presented as median values with IQRs and categorical variables are numbers with percentages.

* Significant difference between survivors and non-survivors.

FiO_2_, fraction of inspired oxygen; LIP, lung injury prediction; NIV, non-invasive ventilation; SpO_2_, plethysmographic oxygen saturation; UVA, universal vital assessment.

**Figure 3:**
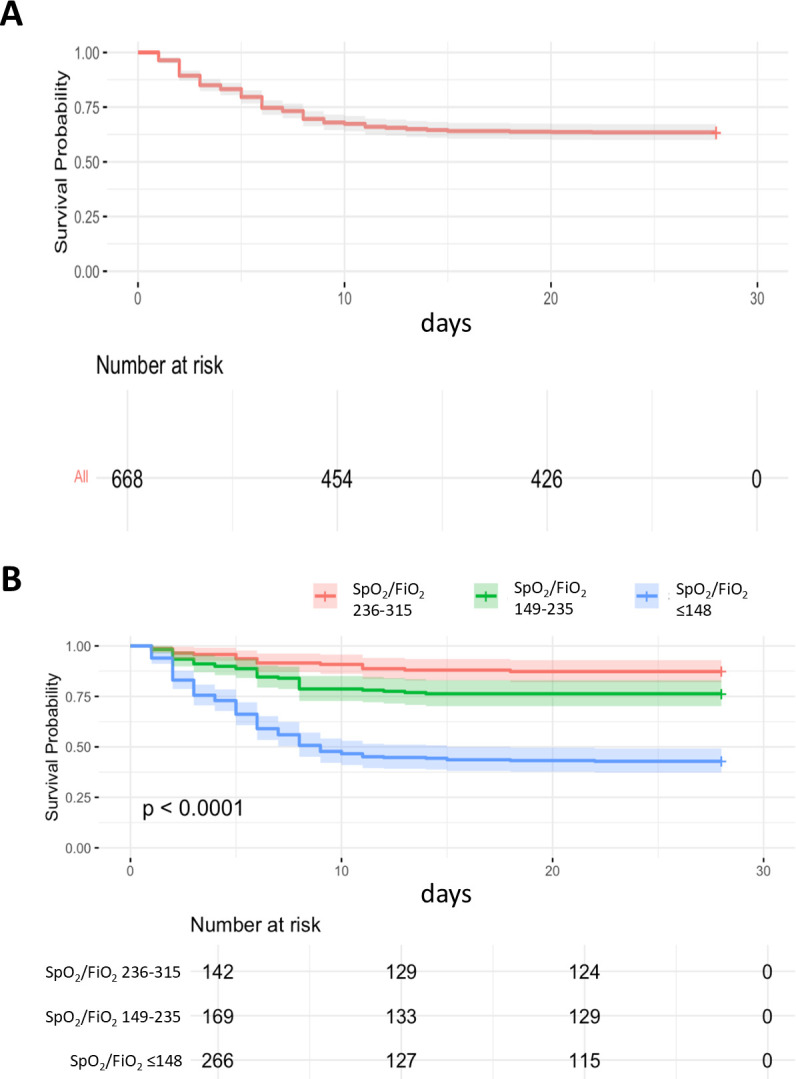
Kaplan–Meier curve of (**A**) all study patients and (**B**) study patients stratified by the severity of hypoxaemia. FiO_2_, fraction of inspired oxygen; SpO_2_, plethysmographic oxygen saturation.

The clinical outcomes of study participants are shown in table 3. Non-survivors were more often admitted to the intensive care unit and underwent invasive mechanical ventilation than survivors. The 28-day all-cause mortality rate in our study population with AHRF was 37.9% (258/682) (figure 3A). 28-day all-cause mortality rates significantly differed between patients when categorised by the severity of hypoxaemia at hospital admission (figure 3B).

**Table 3:** Clinical outcomes of all study participants, survivors and non-survivors

		Total	Survivors	Non-survivors	P value
N		682	424	258	
ICU admission	n (%)	38 (5.6)	8 (1.9)	30 (11.6)	<0.001*
Invasive mechanical ventilation	n (%)	38 (5.6)	8 (1.9)	30 (11.6)	<0.001*
Hospital length of stay	days	5 (2–8)	4 (2–8)	5 (2–8)	0.28
28-day all-cause mortality	n (%)	258 (37.9)	–	–	–

Continuous variables are presented as median values with IQRs and categorical variables as numbers with percentages.

* Significant difference between survivors and non-survivors.

ICU, intensive care unit.

In the multivariate logistic regression analysis, independent risk factors for death (all collected at study entry) included the plethysmographic oxygen saturation (adjusted relative risk (aRR) 1.06 (95% CI 1.02 to 1.09); p=0.001), the lung injury prediction score (aRR 1.83 (95% CI 1.43 to 2.36); p<0.001), respiratory rate>30 breaths per minute (aRR 2.39 (95% CI 1.34 to 4.26); p=0.003) and age>65 years (aRR 2.09 (95% CI 1.13 to 2.86); p=0.02) (table 4).

**Table 4:** Results of a binary logistic regression analysis to predict 28-day mortality

Risk factors (Collected at study entry)	Units	aRR (95% CI)	P value
LIP score	points	1.83 (1.43 to 2.36)	<0.001*
SpO_2_	%	1.06 (1.02 to 1.09)	0.001*
Respiratory rate>30 breaths per minute	binary	2.39 (1.34 to 4.26)	0.003*
Age>65 years	binary	2.09 (1.13 to 2.86)	0.02*
UVA score	points	1.14 (1 to 1.29)	0.05
COVID-19	binary	0.87 (0.48 to 1.58)	0.64
Mean blood pressure	mm Hg	1 (0.98 to 1.01)	0.69

* Significant independent association with 28-day mortality.

aRR, adjusted relative risk; LIP, lung injury prediction; SpO_2_, plethysmographic oxygen saturation; UVA, universal vital assessment.

## Discussion

Our study is the first multicentre study to explore the prevalence, epidemiology and clinical outcomes of AHRF among adults requiring unplanned hospitalisation in sub-Sahara Africa. The prevalence of AHRF in our cohort was almost identical to the prevalence of AHRF we previously reported in a previous single-centre study from Uganda (4.5%)^1^ and another single-centre study from neighbouring Rwanda (4%).^13^ Pulmonary and extrapulmonary infections were the most common causes of AHRF. The 28-day all-cause mortality of participants with AHRF was 37.8%. The severity of hypoxaemia, respiratory rates >30 breaths per minute and the lung injury prediction score (all collected at study entry), as well as age>65 years, were independently associated with mortality from AHRF.

More than half of the AHRF cases in our cohort were caused by pulmonary infection. Since a thoracic radiograph could not be obtained in all study participants due to its inconsistent availability, we were unable to determine the prevalence of pneumonia or ARDS among our cohort. The high rate of pulmonary infections in our study population reflects the high burden of infectious diseases as a cause of acute and critical illness in Uganda^14^ as well as other sub-Saharan African countries.^15 16^ Furthermore, our findings are in keeping with results from previous African and international studies on the epidemiology of AHRF or ARDS.^1 13^ In our previous single-centre study, including critically ill patients admitted to the Ugandan National Referral Hospital in Kampala, we observed that community-acquired pneumonia was the cause of AHRF in 80% of cases.^1^ In another single-centre study undertaken in neighbouring Rwanda, pulmonary infections were reported as the clinical insult predisposing to ARDS in 25% of the patients.^13^ The international LUNG-SAFE study, which included patients with ARDS from 459 intensive care units in >50 countries of mixed-income status, similarly identified pneumonia as the most common cause of ARDS worldwide (59% of cases).^3^

Extrapulmonary infections were the second most common cause of AHRF in our study. Sepsis due to extrapulmonary causes is a leading cause of death among patients requiring unplanned hospitalised, especially those with acute respiratory failure.^17 18^ Further causes of AHRF in our cohort were pulmonary oedema and stroke leading to tracheal aspiration, which are also well-recognised causes of AHRF in high-income countries.^19^,^21^ Approximately, one-thirds of the underlying causes considered to have led to AHRF in our cohort were due to non-communicable diseases, which may reflect the steady increase in the incidence of (unrecognised and untreated) non-communicable diseases, such as arterial hypertension, diabetes mellitus and congestive heart failure among the Ugandan and African populations.^22^ The fact that almost two-thirds of our study cohort suffered from at least one comorbid condition further underlines this result.

The 28-day all-cause mortality rate of AHRF in our study cohort (37.8%) was higher than that reported in high-income countries. The rate of intensive care unit admission in our study population was very low (5.6%). Given that invasive mechanical ventilation has been shown to improve the odds of survival, even when performed in a resource-limited setting,^23^ restricted access to invasive mechanical ventilation (eg, as a result of the very low intensive care unit capacity of the study hospitals) is likely to have contributed to the high mortality observed in our study. Nonetheless, the mortality rate of our population was similar to the hospital mortality (35%) reported by a recent multicentre study evaluating the impact of hypoxaemia among adults admitted to five hospitals in sub-Saharan Africa.^24^ It compared favourably to that reported by other African studies evaluating the prevalence and outcome of AHRF (hospital mortality, 77%),^1^ acute respiratory failure (ICU mortality, 54.5%)^23^ and ARDS (hospital mortality, 50%).^13^ Increased resource allocations by the government in response to the COVID pandemic may have improved the management of patients with all causes of AHRF.

The degree of respiratory compromise at hospital admission, as reflected by the severity of hypoxemia and high respiratory rates, was an important independent risk factor of 28-day mortality in our study population. In view of recent data highlighting a reduced accuracy of plethysmographic oximetry measurements in dark-skinned patients, particularly at lower oxygen saturations,^25^ it is conceivable that the degree of hypoxaemia may even have been underestimated in our study. The pulse oximeters used in our study were commercial products, not explicitly calibrated for research purposes. This, and the study population, may further have contributed to the inaccuracy of measurement.

Our study has several strengths. First, using a multicentre design, it enrolled participants admitted to 11 public hospitals in Uganda. This increases the generalisability of our results to other public hospitals in the country. Second, our study included 682 participants with AHRF, which is an adequate sample size for the questions we address. Third, the 10-month observation period covered both dry and rainy seasons, thus attenuating the seasonal variations in the prevalence of AHRF. Finally, its observational nature, without study-related data, allowed us to document the real-world data regarding the clinical course and outcomes of adult patients hospitalised due to AHRF in Uganda.

When interpreting the results of our study, it is important to consider that the COVID-19 pandemic may have relevantly altered both clinical practice and health system dynamics during the study period. It is likely that the pandemic influenced the prevalence of severe respiratory illnesses, potentially inflating the apparent burden of acute AHRF compared with prepandemic periods. Additionally, the wider availability of pulse oximeters in the country, as catalysed by COVID-19 protocols, likely increased the detection rates of hypoxaemia and may have introduced a detection bias into this observational study. This could have diluted the overall severity of AHRF in our cohort. Furthermore, during the COVID-19 pandemic, the publicly funded healthcare systems of Africa, including Uganda, experienced substantial disruptions in service delivery for non-respiratory conditions, with many patients avoiding care because of fear from infections or due to reallocation of healthcare resources.^26^,^28^ As a result, the population of hospitalised patients during the observation period of this study may have been skewed towards those with respiratory illnesses or COVID-19. These considerations potentially limit the generalisability of our study results to postpandemic periods.

Further limitations should also be highlighted. First, the documented cause of AHRF was based on clinical assessment of the attending physician rather than radiological and laboratory (including microbiological) confirmation. Therefore, we cannot exclude that some causes of AHRF may, thus, have been under- or overestimated. Second, the limited availability of laboratory and radiological services precluded the determination of the prevalence of community-acquired pneumonia and ARDS, limiting the comparability of our findings to other study populations, particularly those from high-income countries.

In conclusion, the incidence of AHRF in adults requiring unplanned hospitalisation in Uganda was 4.1%. Pulmonary and extrapulmonary infections were the most common causes of AHRF, with non-communicable diseases contributing to the underlying aetiology of the remaining causes. The 28-day mortality associated with AHRF (37.9%) was higher than that reported by high-income settings, which may reflect both the higher disease severity at presentation and limited access to advanced organ support such as invasive mechanical ventilation.

## Supplementary material

10.1136/bmjgh-2024-017949 online supplemental file 1

## Data Availability

All data relevant to the study are included in the article or uploaded as supplementary information.
