# Supplementary material for: The epidemiology and outcomes of adults with acute hypoxaemic respiratory failure in a low-income country in the context of the COVID-19 pandemic: a prospective, observational, multicentre cohort study
Source: BMJ Glob Health. 2025 Aug 17;10(8):e017949. doi: 10.1136/bmjgh-2024-017949 (PMC12359528; doi:10.1136/bmjgh-2024-017949)
Supplement: online supplemental file 1 [file bmjgh-10-8-s001.docx]

**Reflexivity statement**

**How does this study address local research and policy priorities?**

The study was specifically designed to determine the prevalence and outcomes from acute hypoxaemic respiratory failure in sub-Saharan Africa

**How were local researchers involved in study design?**

The study was primarily conceptualised and designed by AK (based in Uganda) and CS (based in UK) as part of AK’s PhD studies, for which CS is joint supervisor. DPK, JN, NK, MJ, LN and SC all contributed to the conceptualisation of the study.

**How has funding been used to support the local research team(s)?**

The study was supported by funding awarded to AK/Makerere University, Uganda to support AK’s PhD studies. AK is supported through the DELTAS Africa Initiative grant #DEL-15-011 to THRiVE-2. The DELTAS Africa Initiative is an independent funding scheme of the African Academy of Sciences (AAS)’s Alliance for Accelerating Excellence in Science in Africa (AESA) and supported by the New Partnership for Africa’s Development Planning and Coordinating Agency (NEPAD Agency), with funding from the Wellcome Trust grants 107742/Z/15/Z and 222403/Z/21/Z, and the UK government. Additional support was obtained from the Mahidol Oxford Research Unit (MORU), the Makerere Research and Innovation Fund and the Ministry of Health Uganda. CS received no specific funding to support her participation in this work, and was funded by grants provided to her UK-based research programme.

**How are research staff who conducted data collection acknowledged?**

Data collection was undertaken at the eleven research sites, led by AK. The sit lead for all eleven sites is included in the authorship of the manuscript (DK, CoS, DO, JPO, MGN, DK, MB, EO, GK, BJK, LN).

**How have members of the research partnership been provided with access to study data?**

The data are held at Makerere University in Uganda and are available to all members of the research team.

**How were data used to develop analytical skills within the partnership?**

The study was undertaken by AK as part of his doctoral (PhD) research training during which he was supported by CS and MD to develop his analytical skills. Data cleaning and analysis were undertaken by AK, CS, and MD. PBK, CO and HKB verified the data.

**How have research partners collaborated in interpreting study data?**

The interpretation of the data has been led by AK in collaboration with the research site leads. CS contributed to the interpretation of the data.

**How were research partners supported to develop writing skills?**

The initial manuscript draft was co-written by AK and CS as part of his doctoral studies. He was supported to develop his writing skills via workshops provided as part of his doctoral training programme, and via feedback from CS during the drafting process. All authors subsequently contributed to the drafting of later versions and critical revision of the manuscript.

**How will research products be shared to address local needs?**

The manuscript will be published as open access (funded by CS as the Ugandan researchers do not have access to fund to support these costs), which will support the highlighting of the issue of acute hypoxaemia as an important public health issue in Uganda, and the recognition of the variations in infrastructure across Africa (previous data generation in Kigal, Rwanda differs substantially in the available resources). The data are held at the Makerere University, Uganda and are available to all members of the research team for further analysis.

**How is the leadership, contribution and ownership of this work by LMIC researchers recognised within the authorship?**

The first and corresponding author is AK, who is based in Uganda and undertook the work as part of his PhD research. Of the twenty-three co-authors, only two of the authors are based outside Uganda: MD is based in Austria and is a co-author as he contributed to data analysis and training AK in analytical methods. CS is the last author as she provided research supervision for the study, and conceived and developed the study with AK.

**How have early career researchers across the partnership been included within the authorship team?**

The study was undertaken as part of AK’s PhD studies (his PhD is based at Makerere University, Uganda).

**How has gender balance been addressed within the authorship?**

One third of the co-authors, including the last author) are women.

**How has the project contributed to training of LMIC researchers?**

The project was undertaken as part of AK’s PhD studies.

**How has the project contributed to improvements in local infrastructure?**

The data presented demonstrate for the first time the impact of acute hypoxaemia across Uganda, and highlight areas where resource availability may be impacting clinical outcomes, allowing these needs to be subsequently addressed on the basis of data.

**What safeguarding procedures were used to protect local study participants and researchers?**

The study was reviewed and approved by the ethics committee of the Makerere University College of Health Sciences, School of Biomedical Sciences, in Kampala/Uganda (Reference: SBS-699). The project was also peer-reviewed as part of the process of AK applying for the funding to support his PhD research studies.
